# Re-estimation of basic reproduction number of COVID-19 based on the epidemic curve by symptom onset date

**DOI:** 10.1017/S0950268821000431

**Published:** 2021-02-22

**Authors:** K. Hong, S. J. Yum, J. H. Kim, B. C. Chun

**Affiliations:** Department of Preventive Medicine, Korea University College of Medicine, Seoul, Republic of Korea

**Keywords:** coronavirus, basic reproductive number, generation time, epidemic curve, Korea

## Abstract

Previous studies have reported the basic reproduction number (*R*_0_) of coronavirus disease from publicly reported data that lack information such as onset of symptoms, presence of importations or known super-spreading events. Using data from the Republic of Korea, we illustrated how estimates of *R*_0_ can be biased and provided improved estimates with more detailed data. We used COVID-19 contact trace system in Korea, which can provide symptom onset date and also serial intervals between contacted people. The total *R*_0_ was estimated as 2.10 (95% confidence interval (CI) 1.84–2.42). Also, early transmission of COVID-19 differed by regional or social behaviours of the population. Regions affected by a specific church cluster, which showed a rapid and silent transmission under non-official religious meetings, had a higher *R*_0_ of 2.40 (95% CI 2.08–2.77).

The epidemic characteristics of coronavirus disease (COVID-19) are reported by many countries [1–6]; however, the basic reproduction number (*R*_0_) of COVID-19 should be discussed with some concerns. First, the symptom onset date should be used to estimate *R*_0_ mathematically, instead of the date of the positive test result for accurate estimation but many researchers used the diagnosed date as a proxy [3]. In addition, imported cases among confirmed cases should be identified and excluded from the calculation. Finally, it is better to use the mean generation time of COVID-19 measured through a contact trace system [7] by investigating concise contact relationships between cases, not by assumptions or imputations. Since previous studies lacked the abovementioned features, this study aimed to re-estimate the *R*_0_ of COVID-19 through the epidemic curve of early COVID-19 transmission using the measured serial intervals, which is defined as the time interval between the onset dates of symptom(s) in the infector and the infectee, and symptom onset date of not-imported cases.

## Data source and analysis

Data regarding the study population were derived from the COVID-19 surveillance system in Korea Centers for Disease Control and Prevention (KCDC) [7], collected by the COVID-19 National Emergency Response Center, Epidemiology, and Case Management Team. All reported symptomatic cases in the Republic of Korea from 20 January (the date of the first recorded case) until 3 August were enrolled in this study. Symptomatic cases were defined as having respiratory or systemic symptoms of COVID-19 and were confirmed by real-time reverse transcriptase-polymerase chain reaction (RT-PCR) tests [8]. Symptom onset date was defined as the first date of any symptom newly started within 1 week before the diagnosis. By investigating former cases of confirmed cases, we matched 1567 symptomatic infector–infectee pairs which are relevant, to measure the mean serial interval of the population. Personal information was deidentified before the analysis. Since the data were collected as part of rapid response disease control by the government, the requirement of institutional review board approval was waived.

After collecting the frequencies of symptomatic cases daily, we used the ‘*R*0’ package of R version 3.4.2 [9] to create a distribution of generation times and estimate the *R*_0_ using the exponential growth method, which is one of the popular methods used in infectious disease dynamics studies when the growth rate and the serial interval are available in the data [10]. Estimated *R*_0_ as total and as four different regions, Daegu, Gyeongbuk, Gyeonggi and Seoul, are reported since they showed early epidemics in Korea. Since one large specific church cluster (SCC) in Daegu showed rapid and silent transmission based on broad but non-official religious meetings, they are reported separately [3].

## Characteristics of the population and an epidemic curve

Of the 14 423 confirmed cases in the Republic of Korea until 3 August, 10 870 (75.38%) cases were symptomatic. In total, 1347 cases were excluded because they were classified as imported cases. The mean age in the remaining 9526 cases was 46.22 (standard deviation (s.d.): 19.72) years. The mean age in SCC cases was 40.11 (s.d.: 17.22) years, while that in without SCC cases was 51.43 (s.d.: 20.21) years. Men accounted for 3841 (40.32%) overall cases, 1514 (39.42%) SCC cases and 2327 (60.58%) other cases. Women accounted for 5686 (59.68%) overall cases, 2871 (50.49%) SCC cases and 2815 (49.51%) other cases.

**Fig. 1.**
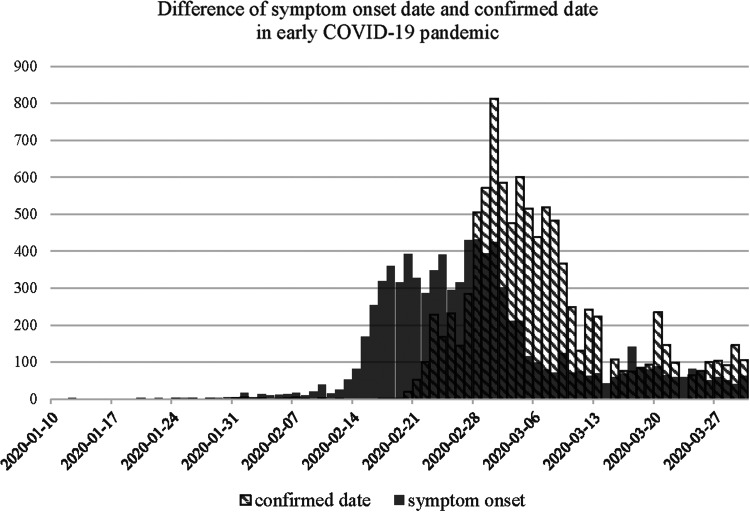
An epidemic curve showing the difference in the distribution of symptom onset date and confirmed date in early COVID-19 pandemic.

Figure 1 shows the difference in epidemic curves by the date used to display frequency. Confirmed date of cases is congregated after the large screening of SCC in late February, which is hard to use as estimating *R*_0_ by the curve. However, symptom onset dates, which is actually used in the *R*_0_ estimation [10], show exponential and much earlier growth than the reported dates. Epidemic curves by regions that showed early transmission of COVID-19 in Korea are shown in Supplementary Figure S1.

## Estimated *R*_0_ of COVID-19

The mean serial interval of 4.02 (s.d.: 4.92) days was fitted into *γ* distribution, which was calculated by symptomatic infector–infectee pairs including 969 infectors and 1567 infectees in the Republic of Korea until 3 August 2020. The total *R*_0_ was estimated as 2.10 (95% confidence interval (CI) 1.84–2.42). SCC cases had an *R*_0_ of 2.40 (95% CI 2.08–2.77) and non-SCC cases had an *R*_0_ of 1.92 (95% CI 1.42–2.59). Regional differences in *R*_0_ are shown in Table 1. Seoul, which was not affected by SCCs, had an *R*_0_ of 1.76 (95% CI 1.20–2.54). Daegu and Geyongbuk showed different *R*_0_ values, regardless of SCC cases; SCC cases in Daegu and Geyongbuk had a higher *R*_0_ than overall of 2.49 (95% CI 2.14–2.91) and 2.37 (95% CI 1.32–4.22), respectively. If we use the publicly reported dates instead of the onset date for the comparison, *R*_0_ for overall was estimated as 3.14 (95% CI 3.04–3.25).

**Table 1.** Basic reproduction number of coronavirus according to the regions

Region	*R*_0_ (95% CI)			Number of population
	Overall	SCC	Without SCC	
Total	2.10 (1.84–2.42)	2.40 (2.08–2.77)	1.92 (1.42–2.59)	9526
Daegu	2.41 (2.14–2.74)	2.49 (2.14–2.91)	1.78 (1.22–2.57)	5473
Gyeongbuk	2.07 (1.55–2.84)	2.37 (1.32–4.22)	1.75 (1.39–2.24)	1290
Gyeonggi	1.53 (1.03–2.18)	–	1.43 (0.99–2.01)	905
Seoul	1.76 (1.20–2.54)	–	1.26 (1.00–1.56)	940

CI, confidence interval; SCC, specific church cluster that showed rapid and silent transmission in the Republic of Korea.

## Discussion

The study showed that the *R*_0_ was 2.10 (95% CI 1.84–2.42). This is lower than 3.14, which is estimated by the reported dates in the Republic of Korea for the comparison. Although estimations through mathematical models or confirmed dates may have advantages in pandemic situation when the onset date is not available [1–6], it is meaningful that these two values may be different because of the following reasons. First, imported cases for the Republic of Korea were 9.34% of total confirmed cases since we had minimal border control. If daily frequencies of COVID-19 were considered including them, transmission rate or *R*_0_ might have been overestimated. Also, due to rapid transmission of SCC group, all members of SCC group were tested for screening, resulting in congestion of confirmed dates which was the date they were tested. Similarly, there was some screening of hospitals also. These tests may have led to an overestimation of *R*_0_ by estimating the larger growth rate than the reality. Therefore, using symptom onset dates is essential for accuracy. Nevertheless, if patients' date of onset is not available, there should be some alternative methods [3] to adjust the estimation gap between the symptom onset and the disease confirmation for the accurate epidemiology of the disease.

Higher *R*_0_ values were estimated in SCC cases, which caused silent transmission in Daegu and Gyeongbuk as confirmations were delayed by uncooperative behaviours of the religious group [4]. The difference in the estimated *R*_0_ by region indicates that the transmission characteristics of COVID-19 may differ based on various factors such as social distancing guidelines or specific clusters showing different characteristics to the general population.

In conclusion, this study estimated *R*0 values ranging from 1.53 to 2.41 in different regions using only the onset date of domestic symptomatic cases. We suggest that the countries should not only report a case by date of symptom onset but also distinguish imported or local transmission, and perhaps even large clusters should be highlighted. Otherwise, estimates of *R*_0_ may be higher than reality.

## Data Availability

Data used in this study are available under special requests to the Korea Centers for Disease Control and Prevention (contact number: +82-043-719-7974).

## Supplementary material

For supplementary material accompanying this paper visit https://doi.org/10.1017/S0950268821000431. click here to view supplementary material
