# Supplementary material for: Re-estimation of basic reproduction number of COVID-19 based on the epidemic curve by symptom onset date
Source: Epidemiol Infect. 2021 Feb 22;149:e53. doi: 10.1017/S0950268821000431 (PMC7925979; doi:10.1017/S0950268821000431)
Supplement: Supplementary file 1 [file S0950268821000431sup.zip › S0950268821000431sup002.docx]

*Epidemiology and Infection*

**Title:** **Re-estimation of basic reproduction number of COVID-19 based on the epidemic curve by symptom onset date**

**Authors:** K. Hong, S.J. Yum, J.H. Kim, B.C. Chun

**Supplementary Material**

Supplementary Figure S1. Epidemic curves by regions that had early epidemic outbreaks in Republic of Korea

(a) Daegu (b) Gyeongbuk (c) Gyeonggi (d) Seoul
