# Supplementary figures and images for: Re-estimation of basic reproduction number of COVID-19 based on the epidemic curve by symptom onset date
Source: Epidemiol Infect. 2021 Feb 22;149:e53. doi: 10.1017/S0950268821000431 (PMC7925979; doi:10.1017/S0950268821000431)

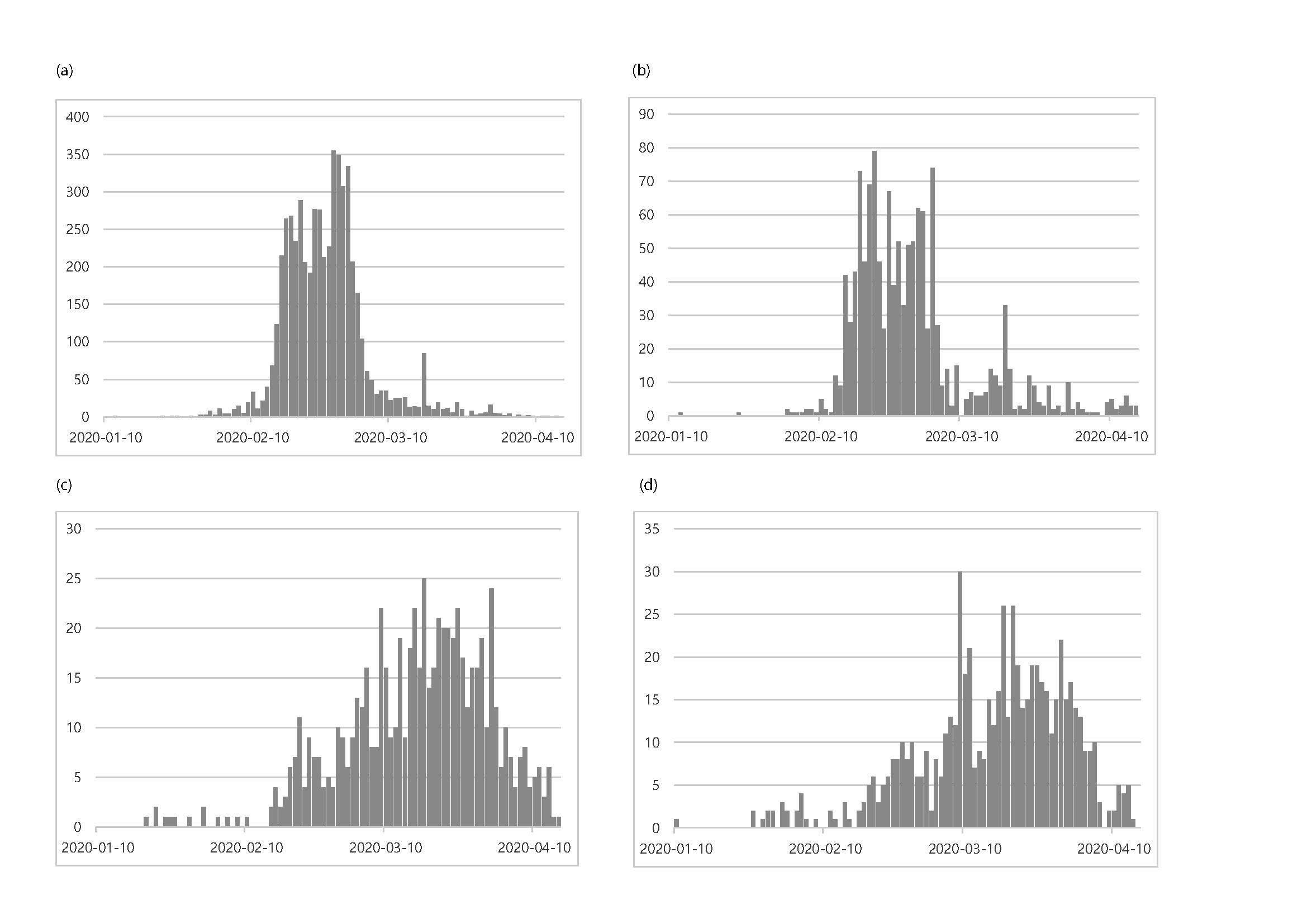

Supplement: Supplementary file 1 [file S0950268821000431sup.zip › S0950268821000431sup001.tif]
